# Biogeography of ocean acidification: Differential field performance of transplanted mussels to upwelling-driven variation in carbonate chemistry

**DOI:** 10.1371/journal.pone.0234075

**Published:** 2020-07-17

**Authors:** Jeremy M. Rose, Carol A. Blanchette, Francis Chan, Tarik C. Gouhier, Peter T. Raimondi, Eric Sanford, Bruce A. Menge

**Affiliations:** 1 Department of Integrative Biology, Oregon State University, Corvallis, Oregon, United States of America; 2 Marine Science Institute, University of California Santa Barbara, Santa Barbara, California, United States of America; 3 Department of Marine and Environmental Sciences, Marine Science Center, Northeastern University, Nahant, Massachusetts, United States of America; 4 Department of Ecology and Evolutionary Biology, University of California Santa Cruz, Santa Cruz, California, United States of America; 5 Bodega Marine Laboratory, University of California Davis, Bodega Bay, California, United States of America; 6 Department of Evolution and Ecology, University of California Davis, Davis, California, United States of America; Brigham Young University, UNITED STATES

## Abstract

Ocean acidification (OA) represents a serious challenge to marine ecosystems. Laboratory studies addressing OA indicate broadly negative effects for marine organisms, particularly those relying on calcification processes. Growing evidence also suggests OA combined with other environmental stressors may be even more deleterious. Scaling these laboratory studies to ecological performance in the field, where environmental heterogeneity may mediate responses, is a critical next step toward understanding OA impacts on natural communities. We leveraged an upwelling-driven pH mosaic along the California Current System to deconstruct the relative influences of pH, ocean temperature, and food availability on seasonal growth, condition and shell thickness of the ecologically dominant intertidal mussel *Mytilus californianus*. In 2011 and 2012, ecological performance of adult mussels from local and commonly sourced populations was measured at 8 rocky intertidal sites between central Oregon and southern California. Sites coincided with a large-scale network of intertidal pH sensors, allowing comparisons among pH and other environmental stressors. Adult California mussel growth and size varied latitudinally among sites and inter-annually, and mean shell thickness index and shell weight growth were reduced with low pH. Surprisingly, shell length growth and the ratio of tissue to shell weight were enhanced, not diminished as expected, by low pH. In contrast, and as expected, shell weight growth and shell thickness were both diminished by low pH, consistent with the idea that OA exposure can compromise shell-dependent defenses against predators or wave forces. We also found that adult mussel shell weight growth and relative tissue mass were negatively associated with increased pH variability. Including local pH conditions with previously documented influences of ocean temperature, food availability, aerial exposure, and origin site enhanced the explanatory power of models describing observed performance differences. Responses of local mussel populations differed from those of a common source population suggesting mussel performance partially depended on genetic or persistent phenotypic differences. In light of prior research showing deleterious effects of low pH on larval mussels, our results suggest a life history transition leading to greater resilience in at least some performance metrics to ocean acidification by adult California mussels. Our data also demonstrate “hot” (more extreme) and “cold” (less extreme) spots in both mussel responses and environmental conditions, a pattern that may enable mitigation approaches in response to future changes in climate.

## Introduction

Awareness of the potential for ocean acidification (OA) to have dramatic impacts on marine ecosystems has exploded in recent decades [1–3]. OA results from increases in atmospheric carbon dioxide (CO_2_) which drives increasing CO_2_ uptake by the oceans with a corresponding reduction in oceanic pH. Secondarily, pH decline reduces carbonate ion (CO_3_^-^) concentration, and can lead to undersaturation, and thus dissolution, of aragonitic calcium carbonate (CaCO_3_) [4,5]. Many marine flora and fauna taxa develop calcified physical features (e.g. shells, tests, skeletons) composed of CaCO_3_. Consequently, research has focused on marine calcifier responses to variation in carbonate chemistry associated with OA [6].

Meta-analyses have summarized laboratory OA experiments on diverse marine taxa, revealing generally deleterious effects for many calcifiers. Among these are negative impacts on calcification, growth, survival, reproduction and abundance [1–3,5,7,8]. OA sensitivity can vary greatly among taxonomic groups; for example crustaceans can show enhanced calcification under OA while molluscs and echinoderms typically show reduced calcification. Differential sensitivities may be trait-mediated [9], and linked to an organism’s developmental stage [1,10–16]. Heavily calcified organisms are generally more susceptible to OA than less-calcified organisms, and active, mobile taxa are generally less sensitive than inactive, sessile taxa [2].

Species responses to OA also can be mediated by other environmental forces. For example, greater resource availability can increase resiliency to OA [3,17,18–22] whereas higher temperatures often exacerbate the effects of OA [2,14,22,23]. Temporal variation in exposure to low-pH conditions also influences organismal responses [24–26].

The complex interplay of abiotic and biotic influences on species responses to OA suggests that there may be ecological “winners” and “losers” in marine communities [4,5,27]. Many calcifying organisms are also strong ecological interactors (e.g., mussels–[28–32]; corals–[33]; oysters–[34]), so OA is likely to have important community-level effects. Our knowledge of such effects is limited, so scaling laboratory results to field ecological performance is therefore critical for furthering understanding of OA impacts on marine communities.

Understanding ecological performance in natural communities requires understanding the local pH environment. Globally, the degree of spatiotemporal variability in pH can vary widely depending on local-scale variation in oceanographic and biological processes [35–37]. Recent discoveries of near-shore marine systems experiencing wider natural variation in carbonate chemistry than forecasted for 2050 in global open oceans [38] offer opportunities for *in situ* studies aimed at understanding organismal responses to OA [33,39–43].

The California Current Large Marine Ecosystem (CCLME) is characterized by wide spatiotemporal variability in nearshore pH conditions [35,44]. As in other Eastern Boundary Upwelled Systems upwelling draws deep dissolved inorganic carbon (DIC) rich, low pH water to the surface, and with further DIC inputs from respiration of dead plankton, results in near-term low-pH and aragonite undersaturation conditions mimicking 50–100 year global ocean forecasts [35,45–48].

### Biological responses

How will coastal marine ecosystems respond to OA? Rocky intertidal systems along the CCLME are heavily populated by calcifying biota, suggesting potential sensitivity of these communities to projected changes in ocean chemistry [43]. Some evidence suggests that many rocky intertidal inhabitants may already possess physiologically plastic traits facilitating tolerance of projected chemical changes [22,46,48–50]. Alternatively, other evidence indicates that many of these organisms may already be near physiological thresholds with minimal capacity to tolerate additional change [27].

Life history stage also can be important. Although OA effects on marine larvae appear mixed [51], many are negatively affected [11,14,34,49]. Negative effects on larvae, however, may not persist. Larvae of the marine mussel *M*. *californianus* were strongly negatively affected by expected future levels of pH [12], but this effect was reversed for juveniles [52]. Further, growth of juvenile *M*. *californianus* and its vulnerability to predation varied in a complicated way with OA, food abundance, and water temperature. In productive but low pH seawater, mussels grew faster and were least vulnerable to whelk predation than in less productive but high pH seawater. These results raise the question of how adult *M*. *californianus* fare under OA conditions.

Along the west coast of North America, *M*. *californianus* is a dominant space competitor, out-competing all other biota [32,53], and providing habitat for diverse taxa [54,55]. Mussels are subject to strong top-down control by the keystone sea star predator, *Pisaster ochraceus* [31,32,56–58], and bottom-up influences from high near-shore productivity [59–61]. As a bivalve mollusc, mussels rely heavily on their calcium carbonate shells for predation resistance, desiccation prevention, resistance of lateral growth pressures, and hydrodynamic force dissipation. Thus, understanding mussel responses to environmental variability can inform understanding rocky intertidal community responses to climate change.

Here, using translocation experiments at 8 sites spanning ~1300 km, we tested the *in situ* mussel performance in relation to environmental conditions. At each site we monitored pH, mussel body temperature and primary productivity. We tested two hypotheses. H_1_: Mussel performance will be negatively affected by lower pH compared to sites experiencing higher pH. H_2_: The influence of pH will be confounded by effects of air and water temperature and phytoplankton productivity, factors that have been shown to contribute to increased growth of *M*. *californianus* [61,62]. We aimed to determine the relative importance of each factor in driving mussel performance, and how these effects varied in space and time.

## Methods

### Study sites

**Fig 1:**
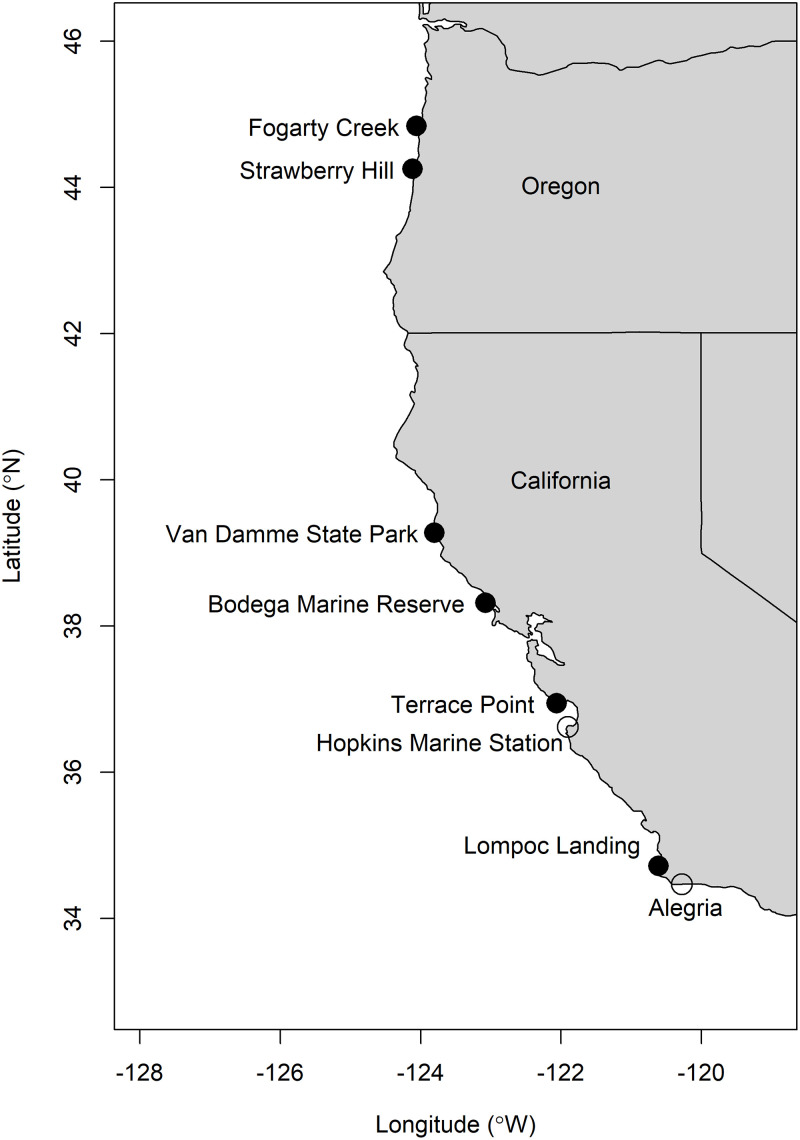
Locations of mussel studies conducted in 2011 and/or 2012. Locations marked with a filled circle (●) indicate sites studied in both years, and those with empty circles (○) indicate sites included in 2012 only. Map made using the R statistics platform [64] using data from US Department of Commerce, Census Bureau, County Boundary File, computer tape, available from Customer Services, Bureau of the Census, Washington DC 20233.

Intertidal sites were distributed across four regions: central Oregon, northern California, Monterey Bay in central California, and southern California (Fig 1, Table 1) that captured the range of environmental variability encountered across this portion of the CCLME. Sites in both 2011 and 2012 included Fogarty Creek and Strawberry Hill in Oregon; Van Damme State Park and Bodega Marine Reserve in northern California; Terrace Point in central California; and Lompoc Landing in southern California. Hopkins Marine Station in central California and Alegria in southern California were added as study sites in 2012. Each site was a wave-exposed rocky intertidal bench with mid-zone *M*. *californianus* beds with biota typical of the CCLME intertidal zone [63]. Detailed descriptions of each site can be found elsewhere [61,62]. All surfaces below the high tide line are public property, so no permissions were required to work at any site but Fogarty Creek, access to which is through private property and which was granted by the property owner. No rocky intertidal species in this study were endangered or protected.

**Table 1:** Locations and years in which mussel performance was measured at intertidal sites.

Location	Years Studied	Latitude	Longitude	Tidal Height of Plots
Fogarty Creek (FC)	2011, 2012	44° 50.3 N	124° 03.6 W	1.1 m
Strawberry Hill (SH)	2011, 2012	44° 15.0 N	124° 06.9 W	1.9 m
Van Damme State Park (VD)	2011, 2012	39° 16.5 N	123° 48.2 W	0.3 m
Bodega Marine Reserve (BM)	2011, 2012	38° 19.1 N	123° 04.4 W	0.3 m
Terrace Point (TP)	2011, 2012	36° 58.6 N	122° 03.9 W	1.3 m
Hopkins Marine Station (HM)	2012	36° 37.2 N	121° 54.5 W	1.3 m
Lompoc Landing (LL)	2011, 2012	34° 43.1 N	120° 36.5 W	1.5 m
Alegria (AL)	2012	34° 28.0 N	120° 16.7 W	0.1 m

### Mussel transplants

Using a standard protocol, performance of California mussels (30–45 mm total length) was quantified during 2011 and 2012 upwelling seasons [61,62,64]. We first collected mussels for pre-study measurements and individual tagging. Under permits from the Oregon Department of Fisheries and Wildlife and the California Department of Fish and Game (Oregon Department of Fish and Wildlife, 2010 permit #15122 and California Department of Fish and Wildlife S-183160003-18316-001), mussels were haphazardly collected from the vertically middle portion of *M*. *californianus* beds. In 2011 but not 2012, to assess genetic or persistent phenotypic influences on mussel performance, we translocated intermingled local-source (i.e., those from each site) and common-source (mussels from a single site, Bob Creek, Oregon, USA). To distinguish them from local-source mussels, common-source mussels were also marked with a bead of epoxy.

In the lab, translocation mussels were marked with a 1–2 mm triangular notch filed on the posterior shell edge (growing lip) to establish an indicator of initial length. Pre-outplant shell weight was estimated using a buoyant-weight method similar to [65]. Briefly, the process involved collecting separate mussel samples for model calibration at Bob Creek, Bodega Marine Reserve (northern California), Sandhill Bluff in central California and Lompoc Landing (southern California). The buoyant weight of each “calibration” mussel was measured by placing the live mussel on a platform submerged in water. The shells of each mussel were pinched closed during transfer through air, to prevent the confounding effect of air intake on buoyancy. Thus, submerged weight was an estimate of the negatively-buoyant shell weight. Soft tissue was then dissected from the shell and, after drying, shell weight was directly measured. The site-specific relationship between buoyant weight and dry shell was modeled using linear regression. The slope and intercept of each model was then used to estimate pre-study shell weight for translocated mussels. The Bob Creek regression model was used for Fogarty Creek and Strawberry Hill mussels, the Bodega Marine Reserve model for Van Damme and Bodega Marine Reserve, the Sandhill Bluff model for Terrace Point and Hopkins Marine Station, and the Lompoc Landing model for Lompoc Landing and Alegria.

#### Mussel translocation

After pre-outplant processing, mussels were translocated back to the field for the April through October upwelling season. In 2011, mussels were sorted into 5 replicate groups of 50 per site, with each group consisting of 25 local- and 25 common-source individuals. For the 2012 season, mussels were sorted into 5 replicate groups of 30 per site.

Mussel translocation used established methods [59]. Briefly, at each site, mussels were placed ventral side down in cleared plots 2–5 m apart within existing mussel beds. Because bed heights varied among sites along the coast, tidal height of transplants varied (Table 1). We accounted for these differences by using tidal height as a covariate in data analyses. Mussels were held in place with plastic mesh (1-cm x 1-cm mesh) that was fastened using stainless steel lag screws inserted into pre-drilled holes with wall anchors. Two to four weeks later, the mesh was loosened to encourage more byssal thread production, and then 2–4 weeks later loosened further into a “dome” to allow space for growth while protecting the mussels from predation.

#### Sample processing and growth measurements

Within 12 hours of collection, all mussels were placed in seawater tables, then within two days of collection, frozen at -20°C. During processing, mussels were thawed, measured (length, width, and depth to the nearest 0.01 mm. Epibionts and byssal threads were removed from the shell exterior, and mussels were then dissected into two constituent parts–shell and soft tissue. These were dried separately at 80°C for ≥ 5 days then weighed to the nearest mg.

Shell-length growth was measured as mm new shell accumulated between the pre-study notch and the growing edge of the shell. Growth was standardized by dividing by initial length. Shell-weight growth was measured as the difference in pre- and post-study shell weight (g), standardized to the individual’s estimated pre-outplant shell weight and the study-season duration at each site.

Shell-weight growth of each mussel was calculated as the difference between the measured dry shell weight at the end of the season and the pre-season shell weight as determined by the previously described buoyant weighting method.

The condition index (unitless) of each mussel was measured as the dry tissue mass per total (tissue + shell) dry mass. Higher condition index mussels have proportionately more soft tissue mass and may reflect energy allocation favoring tissue development [66,67]. Higher condition index may also reflect higher resource quality for mussel predators.

Mean shell thickness index (mg/mm^2^) was estimated by calculating the dry shell mass per shell surface area, with surface area (*A*) calculated by the ellipsoid model *A* = *l* × (*h*^2^ + *w*^2^)^1/2^ × *π* ÷ 2, where *l*, *h*, and *w* are mussel length, height and width, respectively [68]. All shell dimensions were measured to the nearest 0.1 mm, and shell weight was measured to the nearest 0.01g. The resulting index assumes a constant crystalline density of the shell structure. Major predators of *M*. *californianus* include *Nucella* whelks consume mussels through holes drilled their shells. Therefore, mean shell thickness index may correspond to drilling susceptibility [69].

### Environmental characterizations

#### Temperature

Temperature data were obtained using mussel biomimetics, which mimic the thermal properties of living mussels [70,71]. Each logger consisted of a thermistor-based temperature recorder (Tidbit logger, Onset Computer Corp., Bourne, MA) embedded in an epoxy mold shaped like an adult mussel. Using Z-spar epoxy, one to two loggers were deployed per site near replicate mussel plots, then covered with a plastic mesh cage to mimic conditions experienced by the transplanted mussels. Loggers recorded temperatures at 10-minute increments. Air and water temperature data were separated [72] and used to calculate mean temperatures by site and upwelling year.

#### Phytoplankton abundance

Phytoplankton are the primary food of *M*. *californianus* [73]. Food availability was quantified using chlorophyll-*a* concentrations ([Chl-*a*]) as a proxy for phytoplankton abundance. Chl-*a* was measured by periodically collecting water samples in opaque bottles during low tide at each site [74–76]. Replicate (n = 3) bottle samples were collected at low tide from the shore at ~0.5m below the water surface. In the field, fifty ml of water was passed through 25-mm pre-combusted 0.7-μm Whatman GF/F glass-fiber filters. Filters were placed on ice and taken to the lab where Chl-*a* concentrations were quantified using a fluorometer. Because discrete sampling was not consistently conducted at all study sites in both study years, for analysis we averaged all bottle samples across all sample years creating site-specific long term mean summaries of Chl-*a* data. Prior research has shown spatial variability but temporal consistency in the levels of Chl-*a* among subsets of the sites used in this study [74,77].

#### pH measurements

pH data were collected at 10-minute intervals using autonomous sensors deployed at each site within 20 meters from the mussel plots. Sensors were attached to the rock using methods similar to the mussel translocations except that they were held down with stainless steel mesh. Care was taken to ensure that the sensing electrode remained wet even at low tide. Details on these custom-designed sensors can be found elsewhere [49,78], but briefly each was based on an ion-sensitive Honeywell Durafet^®^ with an integrated data logger and power supply [79]. Sensors were calibrated either directly against certified reference materials or indirectly using spectrophotometric pH samples that were calibrated using certified reference materials. pH is reported on the total hydrogen ion concentration scale [80]. Calibrations occurred pre- and post-deployment for all sensors. To spot-check sensor performance, sensor data were periodically (2–4 weeks) compared to discrete water samples collected at all sites except at the two southern California sites in 2012 (Lompoc Landing and Alegria).

To investigate how different aspects of the pH environment might influence mussel performance at each site and in each study year, we compiled summary statistics for the pH mean, standard deviation, and percentages of exposure below two thresholds: pH 7.8 and pH 7.7. These thresholds were chosen for their alignment with model predictions of average global pH conditions by the year 2100 [4,5,6]. Using tide tables, sensor data collected when tides were below the sensor were excluded from analysis.

### Statistical analyses

All analyses used the R statistical package, version 3.5.1 [81]. Mixed-effects models were performed using the lmer function in the lme4 package [82] and hierarchical partitioning was conducted using the hier.part package [83].

We used mixed effects models to assess the spatiotemporal patterns of mussel morphology and performance, with replicate within site as the random intercept for all models. The multiplicative fixed effects of site and year were analyzed at all sites that included mussel responses for 2011 and 2012. Analysis of the multiplicative fixed effects of site and mussel source were limited to 2011 mussel data. Post-hoc pairwise comparisons were estimated using Tukey’s HSD in the R package agricolae [84].

To investigate the effects of seasonal pH, temperature and [Chl-*a*] on variation in mussel performance, we first assessed the most parsimonious additive linear regression model by stepwise AICc model selection using the R function step.AIC [85]. Mussel responses included seasonal growth in shell length and shell weight, condition index and mean shell thickness index. Because this method assumes independence among the explanatory variables, we also examined the Pearson correlation coefficients among these variables.

When collinearity of environmental aspects was detected, we employed hierarchical partitioning to derive the independent effects of each explanatory variable [86,87]. Hierarchical partitioning involves a series of iterative comparisons that assesses the increase in linear fit that results from including a given explanatory variable in an additive model compared to the same additive model without the given variable [87,88]. As an example with the mean pH variable in our study, this partitioning method first compares the increased fit from the null model of ƒ(∅) to the ƒ(mean pH) model, then from ƒ([Chl-*a*]) to ƒ([Chl-*a*] + mean pH), and so on until the added fit has been calculated for all possible linear combinations of explanatory variables with and without the mean pH variable. The series of calculated increase in linear fit are then averaged to provide the independent contribution of the given explanatory variable–mean pH in our example–toward the response variable. This method is particularly amenable to resolving the separate and combined impacts of covarying explanatory variables [89].

## Results

### Spatiotemporal effects

**Fig 2:**
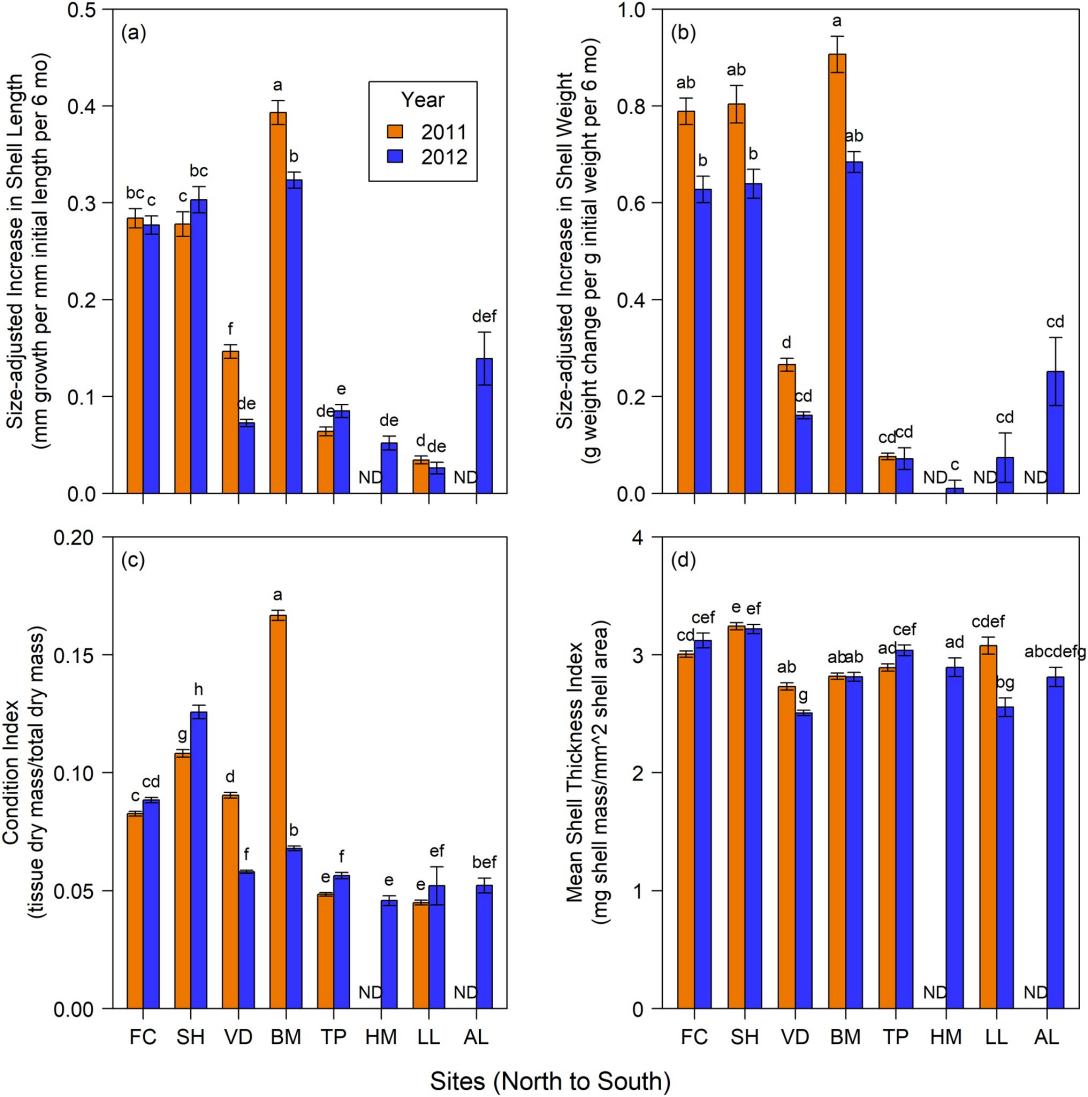
Mussel responses. Mean (±SE) seasonal responses of (a) added shell length, (b) added shell weight, (c) condition index and (d) mean shell thickness index at sites from central Oregon to southern California during the 2011 and 2012 upwelling seasons. Sites are arranged left to right from northernmost to southernmost. Letters indicate differences among sites and/or years (Tukey’s HSD, alpha = 0.05). ND = no data.

At the six intertidal sites studied in 2011 and 2012 (Fig 2; Table 2), mussel performance varied by both site and year (Table 2, site x year interactions). Added length, added weight and condition index were highest at Bodega Marine Reserve in 2011 (Fig 2a–2c; Tukey’s HSD: p<0.0001 for all pairwise interactions between Bodega Marine Reserve in 2011 and other sites). Although variable tide height might explain some of this difference, Alegria mussels were even lower (Table 1) and in 2012 did not grow at rates similar to Bodega Marine Reserve (Fig 2a–2c). Further, shell growth at Bodega Marine Reserve in 2012 was not exceptional, and 2012 Condition-Index highs were observed at the two northerly Oregon sites of Fogarty Creek and Strawberry Hill.

**Table 2:** Effects of site and year on mussel responses in both 2011 and 2012. Mixed effects models include site and year as multiplicative fixed effects and replicate as a random intercept.

Response	Source of Variation	Chi-sq	df	P-value
Growth:	Site	820.322	5	**<0.0001**
Shell length	Year	15.492	1	**0.00008**
	Site x Year	61.682	5	**<0.0001**
Growth:	Site	231.792	4	**<0.0001**
Shell weight	Year	14.559	1	**0.00014**
	Site x Year	5.228	4	0.265
Condition Index	Site	964.210	5	**<0.0001**
	Year	381.52	1	**<0.0001**
	Site x Year	2070.69	5	**<0.0001**
Shell Thickness Index	Site	254.213	5	**<0.0001**
	Year	3.485	1	0.062
	Site x Year	84.237	5	**<0.0001**

Excepting Van Damme State Park, growth (shell length and weight) and condition index were higher at northern sites and lower at southern sites plus Van Damme State Park (Fig 2). Although no clear spatial trend occurred in mean shell thickness index, compared to all other sites and years, shells were thickest at Strawberry Hill in both years and thinnest at Van Damme State Park and Lompoc Landing. In 2012, mussels at Van Damme State Park and Lompoc Landing had the thinnest shells among all site and year combinations. Finally, while all responses varied between years (Table 2, main effect of year or site x year interactions), no obvious temporal trend was detected (Fig 2).

### Source effects

**Fig 3:**
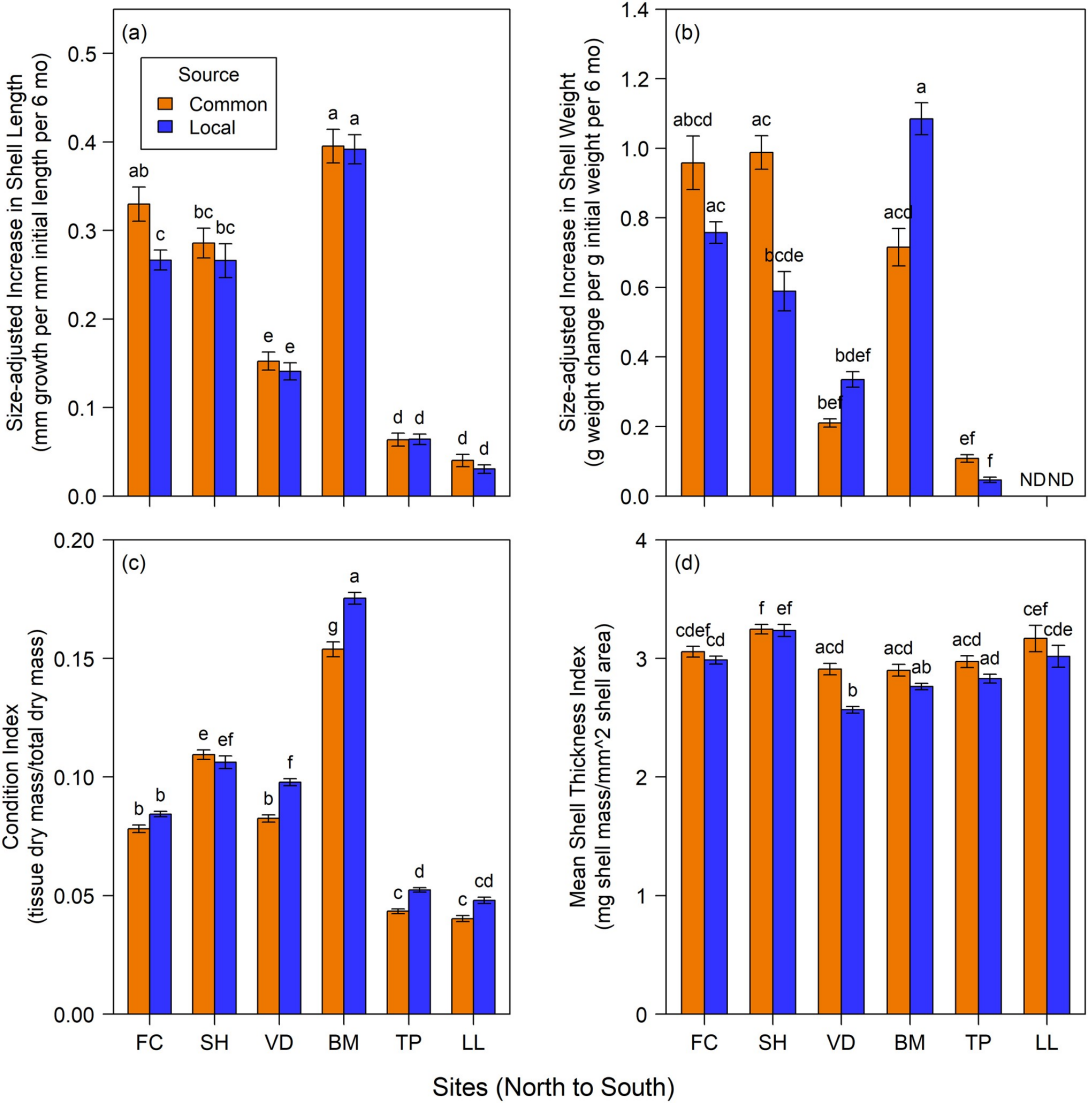
2011 experiments. Mean (±SE) responses of (a) added shell length, (b) added shell weight, (c) condition index and (d) mean shell thickness index between locally-sourced and common-sourced mussels at intertidal sites from central Oregon to southern California. Sites are arranged left to right from northernmost to southernmost. Letters indicate differences among sites and/or years (Tukey’s HSD, alpha = 0.05). ND = no data.

Among the six sites studied in 2011 (Fig 3; Table 3), mussel source (local site source vs. common source of Bob Creek, OR) affected all measures of performance, either as a main effect or through its interaction with site. However, effects were complex. Length of common-source mussels increased faster than that of local-source mussels at Fogarty Creek in Oregon, but source had no effect at any other sites (Fig 3a). Shell weight of common-source mussels tended to increase more for Oregon and Terrace Point mussels but less for Van Damme State Park and Bodega Marine Reserve mussels (Fig 3b). Except for Strawberry Hill, Condition Index was usually higher for local-source mussels (Fig 3c) while at all sites common-source mussels tended to have thicker shells (Fig 3d), but only the difference at Van Damme State Park was significant.

**Table 3:** Effects of study site and mussel source on mussel responses at sites monitored in 2011. Local-source mussels originated from the study site, whereas common-source mussels originated from a single site at Bob Creek, OR. Mixed effects models include site and source as multiplicative fixed effects and replicate as a random intercept.

Response	Source of Variation	Chi-sq	df	P-value
Growth:	Site	678.213	5	**<0.0001**
Shell length	Source	4.5999	1	**0.0320**
	Site x Source	7.3029	5	0.1990
Growth:	Site	175.025	4	**<0.0001**
Shell weight	Source	2.8839	1	0.0895
	Site x Source	23.210	4	**0.0001**
Condition Index	Site	2172.10	5	**<0.0001**
	Source	94.099	1	**<0.0001**
	Site x Source	62.288	5	**<0.0001**
Shell Thickness Index	Site	118.922	5	**<0.0001**
	Source	31.358	1	**<0.0001**
	Site x Source	16.915	5	**0.0048**

### Environmental conditions

**Fig 4:**
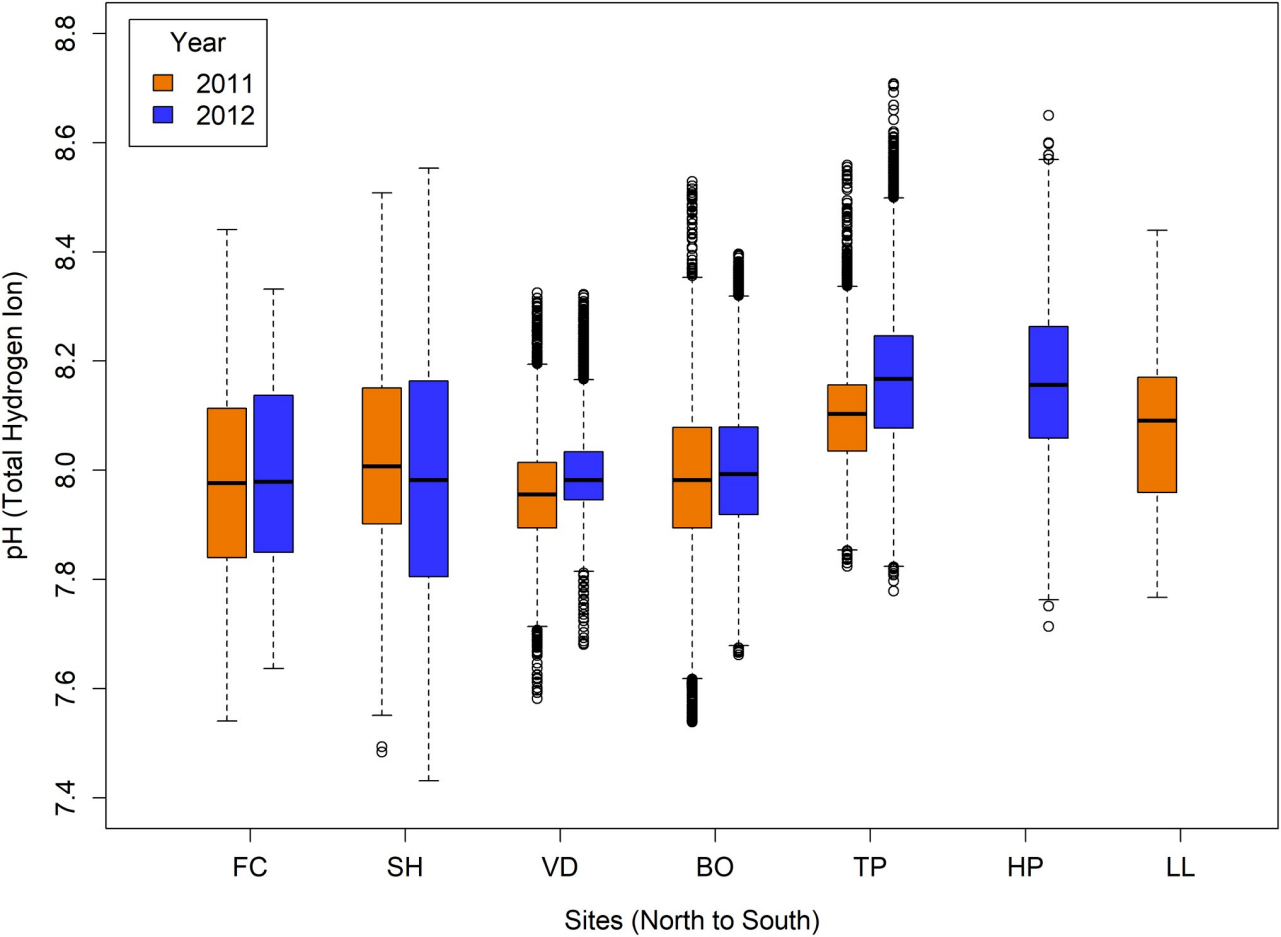
pH in 2011 and 2012. Boxplots showing spatial (among-site) and temporal (2011 and 2012) variation in pH. Sensor data were unavailable from 2011 at Hopkins (HP) and in 2012 at Lompoc Landing (LL). Plots show medians (black line), 25^th^ and 75^th^ percentiles (box), minimum and maximum (lines) and outliers (symbols).

pH varied regionally and among sites but tended to be consistent from 2011 to 2012 (Fig 4). Median pH was lower in Oregon and northern California higher in central and southern California. Notably, Oregon sites tended to be more variable than in California, but sites in northern and central California had relatively large numbers of outliers at both high and low pH. The sites bracketing Monterey Bay, Terrace Point and Hopkins had similar levels, while Lompoc Landing just north of Point Conception tended to be intermediate between the central California and northern sites.

**Fig 5:**
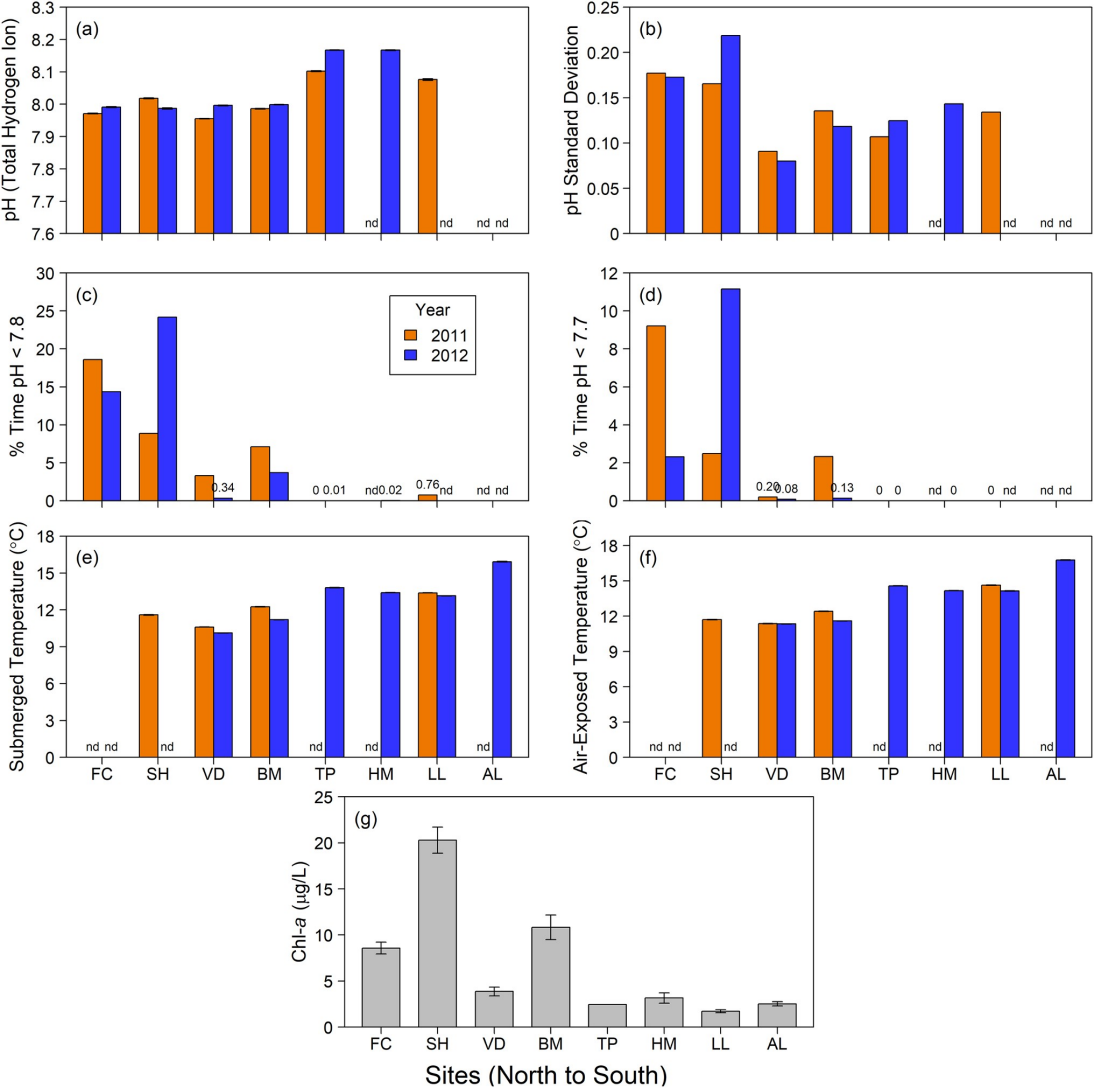
Environmental conditions. Patterns at study sites from north to south (left to right) along the CCLME study region in 2011 and 2012. a. Mean pH, b. mean standard deviation of pH, c. percent of time pH was < 7.8, d. percent of time pH was < 7.7, e. mean temperature of mussel biomimetics when submerged, f. mean temperature of mussel biomimetics when exposed to air at low tide, and g. abundance of chlorophyll-a. nd = no data; when values were 0 or nearly so, the values are shown to distinguish from no data. Panels (a), (e) and (f) include standard error bars, though the small errors result in marginally visible bars.

Environmental conditions for all sites and both study years are summarized in Fig 5. Across both study years, mean pH ranged from a high of 8.17 measured at both Hopkins Marine Station and Terrace Point in 2012 to a low of 7.96 at VD in 2011 (Fig 5a). Comparing between years, the highest mean pH in both 2011 (pH 8.10) and 2012 (pH 8.17) occurred at Terrace Point, while Van Damme State Park recorded the lowest mean pH in 2011 (pH 7.96) and Strawberry Hill recorded the lowest pH in 2012 (pH 7.99). As indicated by Fig 4, variability in pH conditions (mean standard deviation = SD) in both years was greatest at Oregon sites (SD ranged from 0.17 to 0.22) and least at VD (0.08 to 0.09) (Figs 4 and 5b). Mussels at Fogarty Creek and Strawberry Hill also experienced the highest percentage of time exposed to both thresholds of low-pH conditions (ranging from 8.9% to 24.2% [<7.8] and 2.3% to 11.2% [<7.7]) (Fig 5c and 5d). Except for Bodega Marine Reserve (3.7% and 7.1% [pH<7.8], 2.3% [pH<7.7 in 2011]) and Van Damme State Park (3.3% [pH<7.8 in 2011]), all other sites experienced levels of pH as low as 7.8 and 7.7 less than 1% of the time. Lowest temperatures occurred in 2012. Mean mussel body temperature during submersion varied from a high of 15.92°C at southern-most Alegria to a low of 10.13°C at Van Damme State Park (Fig 5f). Similarly, mean mussel body temperature during low-tide exposure varied from a high of 16.77°C at southern-most Alegria in 2012 to a low of 11.34°C at Van Damme State Park (Fig 5f). Because climatological means were used for [Chl-*a*], there were no differences between years in our study. The location with the highest [Chl-*a*] was Strawberry Hill at 20.29 μg/L and the lowest [Chl-*a*] was measured at Lompoc Landing with 1.72 μg/L (Fig 5g).

Correlations among measures of pH indicate that these data were generally interrelated (Table 4). Among pH measures, mean pH was moderately negatively correlated with the pH < 7.8 threshold (Pearson’s r = -0.507), weakly negatively correlated with the pH < 7.7 threshold (r = -0.310), and weakly positively correlated with pH variability (r = 0.369). Variability in pH was moderately positively correlated with both threshold conditions (pH < 7.8: r = 0.568; pH < 7.7: r = 0.652). The two thresholds were strongly positively correlated (r = 0.928).

**Table 4:** Pearson correlation coefficient (r) matrix for environmental measures.

	pH				[Chl-*a*]	Temperature		Tidal height
	Mean	SD	< 7.8	< 7.7		Submerged	Exposed	
pH: Mean	1.000	0.369	-0.507	-0.310	-0.316	0.846	0.879	0.588
pH: SD	0.369	1.000	0.568	0.652	0.659	0.580	0.357	0.771
pH: % < 7.8	-0.507	0.568	1.000	0.928	0.911	-0.217	-0.481	0.164
pH: % < 7.7	-0.310	0.652	0.928	1.000	0.850	-0.028	-0.282	0.265
[Chl-*a*]	-0.316	0.659	0.911	0.850	1.000	-0.190	-0.443	0.355
Temp: Submerged	0.846	0.580	-0.217	-0.028	-0.190	1.000	0.950	0.557
Temp: Air- Exposed	0.879	0.357	-0.481	-0.282	-0.443	0.950	1.000	0.489
Tidal height	0.588	0.771	0.164	0.265	0.355	0.557	0.489	1.000

Measures of pH were also correlated with measures of other environmental factors (Table 4). The strongest positive correlations existed between mean pH and submerged temperature (r = 0.846) and air-exposed temperature (r = 0.879); the pH < 7.8 threshold and [Chl-*a*] (r = 0.911); and the pH < 7.7 threshold and [Chl-*a*] (r = 0.850). The strongest negative correlations existed between mean pH and the pH < 7.8 threshold (r = -0.507); air-exposed temperature and the pH < 7.8 threshold (r = -0.481); and air-exposed temperature and [Chl-*a*] (r = -0.443). The weakest pairwise correlation was between the pH < 7.7 threshold and submerged temperature (r = 0.028).

Among non-pH measures, submerged and air-exposed temperatures were strongly positively correlated (Table 4; r = 0.950). [Chl-*a*] negatively correlated with air-exposed temperature (r = -0.433) and less so with submerged temperature (r = -0.190). Tide height was positively related to all measures of pH, food, and temperature, but most strongly to pH variability.

### Mussel performance responses

**Fig 6:**
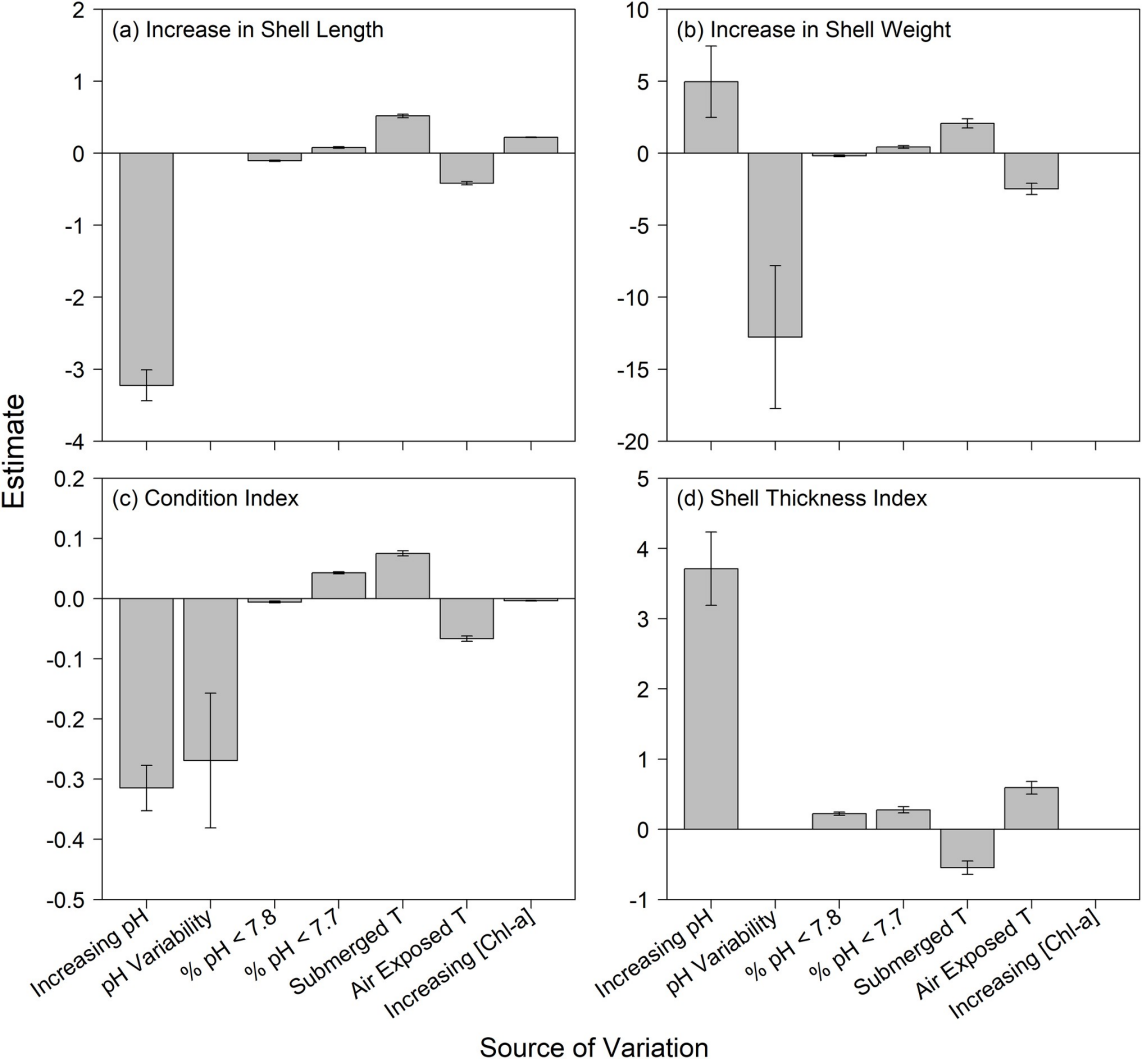
Mussel performance. Sign and magnitude of effect of pH (mean), pH variability (SD), time of exposure to pH < 7.8, time of exposure to pH < 7.7, submergence temperature, air-exposed temperature (low tide), and food availability (abundance of Chl-*a*). Data are the estimate and SE columns from Table 5.

Mussel performance was influenced by all measured environmental factors (four pH measures, two temperature measures, and [Chl-*a*]), but varied among the different metrics (Fig 6, Table 5). Despite the differences among the sites in the level on the shore of the translocations (Table 2), tidal height was not included in the most parsimonious additive linear models for any of the response variables, as indicated through AICc model selection (Table 5). Shell length was influenced by all remaining factors but pH SD, shell weight by all but [Chl-*a*], condition index by all remaining factors, and mean shell thickness index by all but pH SD and [Chl-*a*] (Fig 6, Table 5). Using adjusted R^2^, the rank order of fit for each model was condition index (R^2^-adj = 0.8447), shell-weight growth (R^2^-adj = 0.6092), shell-length growth (R^2^-adj = 0.6011), and mean shell thickness index (R^2^-adj = 0.1874).

**Table 5:** Most parsimonious additive linear models as determined by stepwise AICc for each mussel response.

Response	Source of Variation	Estimate	SE	t value	P-value
Growth: Shell length	Intercept	25.2592	1.6360	15.439	**<0.0001**
(R^2^-adj = 0.6011)	pH: Mean	-3.2240	0.2141	-15.060	**<0.0001**
	pH: % < 7.8	-0.1040	0.0087	-11.952	**<0.0001**
	pH: % < 7.7	0.0790	0.0106	7.432	**<0.0001**
	Temp: Submerged	0.5189	0.0248	20.965	**<0.0001**
	Temp: Air-Exposed	-0.4161	0.0214	-19.455	**<0.0001**
	[Chl-*a*]	0.0221	0.0023	9.628	**<0.0001**
Growth: Shell weight	Intercept	-31.4006	18.2445	-1.721	0.0866
(R^2^-adj = 0.6092)	pH: Mean	4.9692	2.4751	2.008	**0.0459**
	pH: SD	-12.7623	4.9613	-2.572	**0.0107**
	pH: % < 7.8	-0.1831	0.0560	-3.273	**0.0001**
	pH: % < 7.7	0.4383	0.0878	4.999	**<0.0001**
	Temp: Submerged	2.0688	0.3172	6.521	**<0.0001**
	Temp: Air-Exposed	-2.4755	0.3982	-6.217	**<0.0001**
Condition Index	Intercept	2.6048	0.2924	8.909	**<0.0001**
(R^2^-adj = 0.8447)	pH: Mean	-0.3148	0.0375	-8.409	**<0.0001**
	pH: SD	-0.2690	0.1119	-2.405	**0.0164**
	pH: % < 7.8	-0.0058	0.0016	-3.527	**0.0004**
	pH: % < 7.7	0.0428	0.0019	22.731	**<0.0001**
	Temp: Submerged	0.0751	0.0043	17.603	**<0.0001**
	Temp: Air-Exposed	-0.0666	0.0045	-14.741	**<0.0001**
	[Chl-*a*]	-0.0036	0.0005	-7.506	**<0.0001**
Shell Thickness Index	Intercept	28.3960	4.0397	-7.029	**<0.0001**
(R^2^-adj = 0.1874)	pH: Mean	3.7107	0.5227	7.099	**<0.0001**
	pH: % < 7.8	0.2236	0.0227	9.841	**<0.0001**
	pH: % < 7.7	0.2772	0.0443	-6.261	**<0.0001**
	Temp: Submerged	-0.5464	0.0952	-5.738	**<0.0001**
	Temp: Air-Exposed	0.5914	0.0912	6.484	**<0.0001**

Shell length growth and condition index were both higher with low pH (i.e., decreased with increasing mean pH (Fig 6a and 6c), while shell weight growth and condition index were both lower with increasing pH variability (Fig 6b and 6c). In contrast, mean shell thickness index and shell weight growth were both higher with high pH (i.e., with increasing mean pH; Fig 6b and 6d). Exposure time to pH < 7.8 and pH < 7.7 had opposite effects on three measured responses, with negative effects of time < 7.8 and positive effects of time <7.7 on length increase, weight increase and condition index. Exposure time to both pH < 7.7 and pH < 7.8 had positive effects on mean shell thickness index. Impacts of air-exposed and submerged temperature on performance generally mirrored the patterns of pH < 7.8 and pH < 7.7 exposure percentages, respectively. The exception was the negative effect of submerged temperature compared to the positive effect of exposure time to pH < 7.7 on mean shell thickness. Increasing submerged temperature increased length, weight and condition index responses but decreased mean shell thickness index, whereas exposed temperature had the opposite pattern (Fig 6). Increasing food availability measured as [Chl-*a*] positively affected shell-length growth, and negatively affected condition index (Fig 6, Table 5).

**Fig 7:**
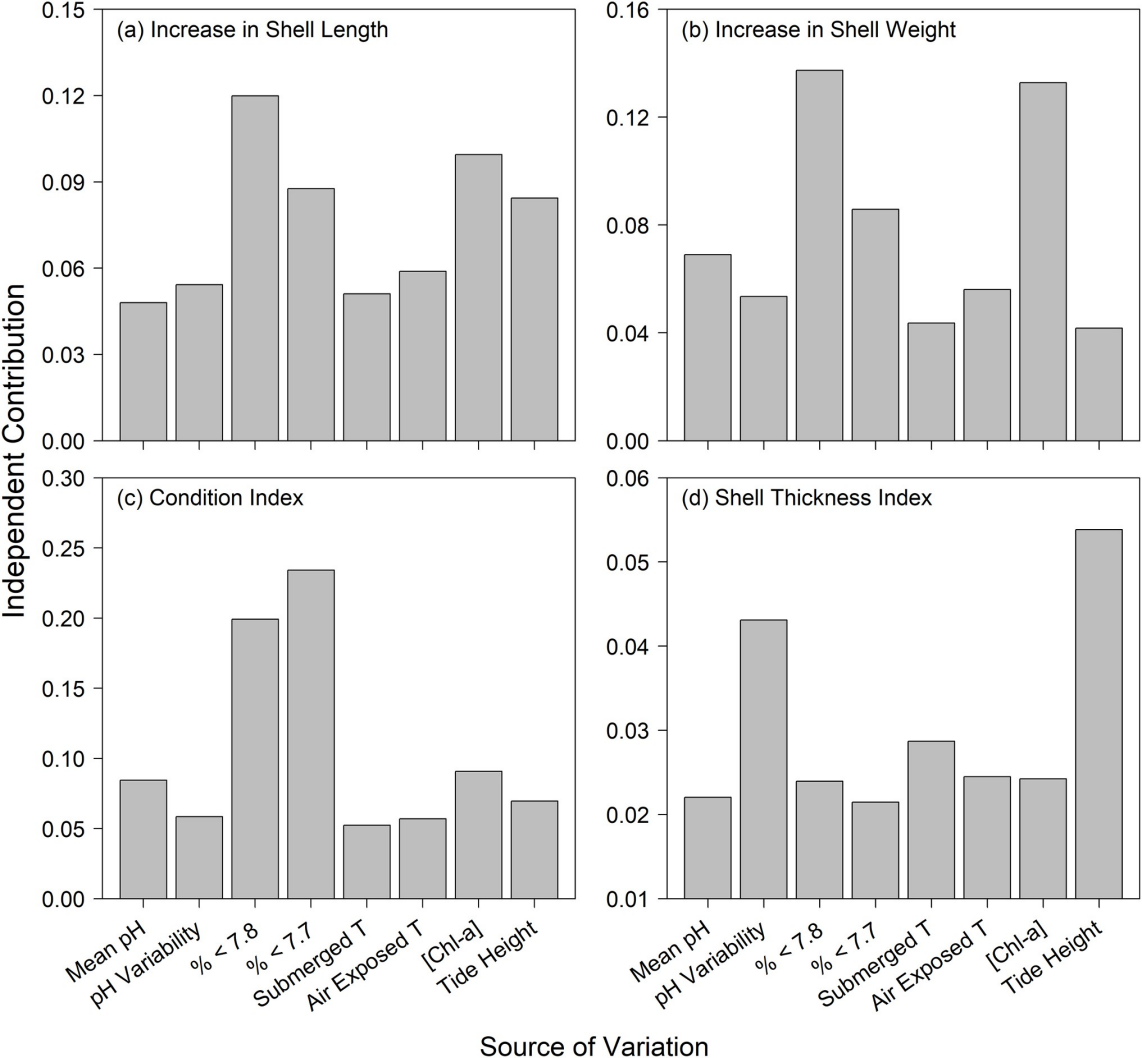
Independent contributions of the predictor variables to (a) shell length growth, (b) shell weight growth, (c) condition index and (d) mean shell thickness index, as estimated from hierarchical partitioning. The predictors for pH include seasonal average (mean) pH, pH variation (SD) and the percent exposure to conditions below the thresholds of pH 7.7 and pH 7.8. The temperature predictors (submerged temperature and air-exposed temperature) are based on seasonal averages, and [Chl-*a*] is based on a long-term climatological summary for each site. Tidal height is the average for all study plots at each site.

In considering all possible models through hierarchical partitioning (Fig 7), of the pH metrics, exposure to extreme low pH (% <7.8, %<7.7) had the strongest effect on length and weight growth measures and on condition index. All four performance responses responded similarly to the independent effects of pH and temperature, while [Chl-*a*] had strong effects on length and weight growth, and tidal height had strong effects on length growth and shell thickness index. pH variability was the primary OA metric associated with shell thickness index.

Both mussel responses and environmental conditions, including pH measures, appeared to vary non-linearly along the coast (Figs 2–5). We tested this possibility by ranking each variable (highest value = 1 to lowest = 7 or 8) and regressing these against site ranks (north = 1 to south = 8). Except for % of time <7.8 pH (p = 0.04) and condition index (p = 0.03; both higher toward the north), and air-exposure and submergence temperatures (p = 0.01; both higher toward the south), no regressions were significant, indicating non-linearity.

## Discussion

Our study revealed that pH conditions were biologically relevant to adult *M*. *californianus* calcified and body condition performance, and the nature of pH influence was mediated by air and submerged temperature and food availability, measured as chlorophyll-*a*. The most surprising result was that, while site-specific correlations of mean pH with mussel shell weight gain and mean shell thickness index supported hypothesis H_1_ of reduced performance under low-pH conditions (i.e., weight and thickness increased most with higher pH), shell length gain and condition index were actually greater with reduced pH. Further, since condition index was affected, pH effects were not limited to the calcification components of *M*. *californianus*. These positive effects of OA on adult mussel shell and tissue growth in the field contrast to the negative effects on larval mussel responses in laboratory studies [12], further supporting the observation that life history can play an important role in organismal responses to OA [49,51]. The complex relationship of pH co-varying with temperature and [Chl-a] highlights the interactive nature of these and other factors in modulating organismal responses to OA [48,52,90].

### Variation in performance

Historically, *M*. *californianus* has exhibited a broad range of ecological performance across the CCLME [61,62,73]. In our study, intertidal seasonal growth varied by as much as ~1500% among sites and between years. Condition index and mean shell thickness index also exhibited ecologically relevant scales of variation among intertidal sites with as much as ~360% and ~180% differences, respectively, among sites and years. Comparisons among overall performances at different sites provide insight into the ecological relevance of these differences. For example, intertidal mussels at Fogarty Creek and Strawberry Hill are generally thick-shelled and meaty (i.e. relatively high condition index) with relatively fast growth compared to mussels at Hopkins Marine Station or Lompoc Landing that have thinner shells, less tissue mass, and much less seasonal growth. Thinner shells increase susceptibility to predation from drilling whelks and reduces the forces required to crush *M*. *californianus* [12,52,91,92]. As filter feeders, mussels also serve a large ecological role in incorporating planktonic carbon into intertidal food webs [73]. This role may be decreased at sites with smaller, less meaty mussels.

### Relative environmental effects

As hypothesized (H_2_), temperature (air-exposed and submerged), [Chl-*a*] and pH all had separate and joint effects on mussel performance along the CCLME. Past studies of *M*. *californianus* along the Oregon coast have found strong positive correlations between mussel growth and [Chl-a] as a proxy for food availability [56,59,76] while the conditions of populations in southern California primarily were linked to temperature, not [Chl-*a*] [62,93]. In contrast to the latter, our results indicated a positive effect of [Chl-*a*] from Oregon to southern California. Our results also agree with prior studies [61,62]–higher submerged and lower air-exposed temperatures correlated with growth in both shell weight and length, and mussel condition index [94].

While we hypothesized that sites exposed to lower and more variable pH would be stressful for adult mussels, as with responses of juvenile *M*. *californianus* [52], our results actually revealed mixed effects of pH on adult compared to young mussels. Consistent with laboratory results that showed that larval mussels grown under elevated CO_2_ had thinner (and weaker) shells and reduced tissue mass relative to control larvae, shell weight and shell thickness index of field-translocated adult mussels were also diminished under conditions of elevated CO_2_ (i.e., were enhanced under lower CO_2_) (Fig 6b and 6d). However, increased shell length and condition index were enhanced, not diminished, under elevated CO_2_ (Fig 6a and 6c). Although pH variability had no influence on increased shell length or mean shell thickness index, both increased shell weight and condition index responded negatively to pH variability.

Negative consequences of increased CO_2_ comparable to those we observed have been observed in other species. For example, the rate of calcium deposition and total weight for juvenile *M*. *chilensis*, and reduced calcification in adult *M*. *edulis* have been documented [22,95]. The direction and magnitude of these results need to be considered with caution given the high degree of collinearity among environmental stressors (e.g., Table 5). Nevertheless, we found that, compared to models that consider only the contributions of temperature and [Chl-*a*] toward explaining variation among mussel responses, adding the independent contributions of pH sufficiently boosted the amount of explained variance to warrant including pH monitoring in future ecological studies of climate stressors.

In considering thresholds of pH exposure, our results indicate that greater exposure below pH 7.8 was associated with reduced increases in shell length and weight, and condition index. This suggests an ecological “tipping point”, below which organisms may increasingly become physiologically stressed. Interestingly, the pattern is switched regarding exposure below pH 7.7. Higher exposure here was associated with enhanced increases in shell length and weight, and condition index. This contradiction may be partially explained by the high correlation between [Chl-*a*] and threshold exposure; high food availability may help compensate for pH stress [21]. Alternatively, the number of events below pH 7.7 may have been relatively few, skewing the direction of the analysis.

These and other apparently contradictory patterns of pH effects among mussel performance metrics highlight the complexities of both environmental variation in the nearshore environment and the interactive effects of multiple environmental stressors on organismal physiology. Robust field-deployable pH sensors have only relatively recently become available for widespread use by the scientific community [79]. Researchers have begun to tease apart the complexities of natural spatiotemporal pH variations [36,95,96] and how such variation coincides with the natural dynamics of other environmental characteristics [97,98]. Such studies are uncovering critical intricacies in natural exposure regimes for marine biota and can greatly inform the nature of future OA laboratory examinations. Laboratory studies that cross variation in other environmental conditions with different pH (or CO_2_) levels are also helping elucidate interactive effects of environmental stressors. For example, when exposed for six months to near-future pH and temperature conditions while undergoing food limitation, adult *M*. *edulis* shell strength was reduced under warming conditions but not by pH [99]. In laboratory and field studies around Kiel Fjord in the Western Baltic Sea, *M*. *edulis* growth and calcification have been shown to be negatively affected by food limitation, but when food is abundant, are resistant to changes in pH [21]. Recent meta-analyses of the available literature found that OA impacts on calcification can be mitigated by food supply [3,17,100]. These and other studies underscore how diverse physiological stressors can have antagonistic or synergistic effects, depending heavily on organismal physiological tolerances [7,9,101].

### Physiological responses

Much research regarding OA effects on calcifying organisms focuses on characteristics of calcification processes, including such responses as calcified growth, net dissolution/calcification and breakage susceptibility [2,102,103]. Mounting evidence indicates that OA stress also impacts other physiological processes [41,42,104–109]. For example, like others [46], we found that pH was an independent and combined contributor toward explaining variance in condition index. These effects on soft tissue may be indicative of an energetic trade-off between shell quality (in terms of growth and mean shell thickness index in our study) and tissue quality or quantity. Analyses using Dynamic Energy Budget (DEB) models indicate metabolic shifts away from growth in favor of maintenance [110,111]. While we did not quantify variation in types of soft tissue, these effects could manifest as reduced musculature and/or gonad production. Studies on adult *M*. *edulis* showing temperature had the greatest impact on performance in food limited conditions hypothesized that the mechanism was a reallocation of limited energy resources toward temperature-related metabolic increases and away from shell strength [99]. O’Donnell et al. [112] found that *M*. *californianus* byssal thread strength decreased under elevated CO_2_ while shell and tissue growth were unaffected. Such detrimental effects of OA on non-calcified structures underscores the importance of investigation of a diversity of physiological responses to OA stress by both calcifiers and non-calcifiers.

### Role of life history stage

Comparisons of responses between mussels from two different source populations at each site in 2011 indicated that performance depended partially on historical environment. Source–that is, whether mussels originated from the local site or from a single common site in Oregon—explained variation in condition index and mean shell thickness index. These results could indicate effects due to genetic or persistent phenotypic differences, which are critically important issues for potential mitigation-related research and for management efforts [113]. Mean shell thickness index did not depend on the year of study, possibly further indicating that thickness is an integrated signal over the life history of the individual. In light of other work showing strong negative effects of pH reductions on mussel larvae [12], our results suggest a transition during life history from susceptibility to OA in larvae to greater resilience to OA in juvenile [52] and adult mussels.

### Hotspots and coldspots along the CCLME

The pH environment along the 1300 km span of the study region previously has been referred to as a “mosaic” of conditions [35,52], and our results indicate the same is true for mussel responses to coastal oceanic conditions. Importantly, the strong associations between mussel performance and environmental conditions (Table 5) and the non-linear patterns across space reveals that mussel performance maps on to the pH regime. That is, this region is characterized by “hot” and “cold” spots, or areas where mussel performance was high (generally to the north of the CCLME), despite seemingly challenging environmental conditions, and low (generally Monterey Bay and southward), despite seemingly more favorable conditions. Although oceanic conditions vary inter-annually, the pattern of variation is consistent through time, potentially enabling mitigation efforts in response to future climate change.

### Conclusions

Our study demonstrates complex responses by *M*. *californianus* to dynamic interconnected variation in temperature, chlorophyll-*a* and pH along the CCLME. Site pH exerted important influences on mussel performance, but these varied in both magnitude and direction depending on response. With some responses, mussels even thrived at sites with lower pH, contrary to the prevailing hypotheses for calcifying biota. Our study underscores the importance of OA investigations that consider real-world ecological contexts in which the impacts of pH variation are likely influenced by other aspects of the natural environment. Organisms that survive and thrive in areas that already undergo variation in pH, such as *M*. *californianus* along the CCLME, may have developed an adaptive capacity to mitigate pH as a physiological stressor given other advantageous aspects of the natural environment.
